# Unveiling the Dynamic Evolution of Catalytic Sites in N-Doped Leaf-like Carbon Frames Embedded with Co Particles for Rechargeable Zn–Air Batteries

**DOI:** 10.3390/molecules29184494

**Published:** 2024-09-22

**Authors:** Yuebin Lian, Weilong Xu, Xiaojiao Du, Yannan Zhang, Weibai Bian, Yuan Liu, Jin Xiao, Likun Xiong, Jirong Bai

**Affiliations:** 1 School of Optoelectronic Engineering, Changzhou Institute of Technology, Changzhou 213032, China; 2 School of Chemistry and Chemical Engineering, Henan University of Technology, Zhengzhou 450001, China; 3 School of Chemistry and Environmental Engineering, Shanghai Institute of Technology, Shanghai 201418, China; 4 Research Center of Secondary Resources and Environment, School of Chemical Engineering and Materials, Changzhou Institute of Technology, Changzhou 213032, China

**Keywords:** electrocatalysts, oxygen evolution reactions, oxygen reduction reactions, Zn–air batteries, metal–organic frameworks

## Abstract

The advancement of cost-effective, high-performance catalysts for both electrochemical oxygen reduction reactions (ORRs) and oxygen evolution reactions (OERs) is crucial for the widespread implementation of metal–air batteries. In this research, we fabricated leaf-like N-doped carbon frames embedded with Co nanoparticles by pyrolyzing a ZIF-L/carbon nanofiber (ZIF-L/CNF) composite. Consequently, the optimized ZIF-L/CNF-700 catalyst exhibit exceptional catalytic activities in both ORRs and OERs, comparable to the benchmark 20 *wt*% Pt/C and RuO_2_. Addressing the issue of diminished cycle performance in the Zn–air battery cycle process, further detailed investigations into the post-electrolytic composition reveal that both the carbon framework and Co nanoparticles undergo partial oxidation during both OERs and ORRs. Owing to the varying local pH on the catalyst surface due to the consumption and generation of OH^−^ by OERs and ORRs, after OERs, the product is reduced-size Co particles, while after ORRs, the product is outer-layer Co(OH)_2_-enveloping Co particles.

## 1. Introduction

Rechargeable zinc–air batteries, due to their cost-effectiveness and exceptional specific energy densities, have garnered significant attention for their potential role in future energy storage and release [1,2,3,4,5]. The performance of these batteries is largely dependent on electrocatalysts that enable both oxygen reduction reactions (ORRs) and oxygen evolution reactions (OERs). At present, the most effective materials for ORRs and OERs are Pt-based and Ru/Ir-based, respectively [6,7,8]. However, the limited availability and high cost of Pt, Ru, and Ir present considerable challenges for their broad application in the development of large-scale rechargeable zinc–air batteries. This has led to extensive research in recent decades that aims to discover high-performance, cost-effective electrocatalysts for ORRs or OERs [9,10,11]. Numerous carbon-based materials and abundant transition-metal-based materials with high inherent activities have emerged as promising alternatives to Pt- or Ru/Ir-based electrocatalysts [12,13,14,15].

These alternatives are particularly effective in alkaline conditions, the typical electrolyte environment in rechargeable zinc–air batteries. Intriguingly, the development of cost-effective, bifunctional electrocatalysts that can proficiently facilitate both ORRs and OERs has been somewhat overlooked [16,17,18,19]. The advent of such bifunctional electrocatalysts could notably bolster the construction of large-scale rechargeable zinc–air batteries [20,21,22]. A prevalent approach to fabricating high-performance multifunctional electrocatalysts involves utilizing metal–organic frameworks (MOFs) as a structural template to construct porous carbon nanostructures [23,24,25,26,27,28]. These structures incorporate active elements such as metal oxides [29,30], phosphides [31,32,33], sulfides [34,35,36,37,38], and even atomically dispersed metal–nitrogen complexes [39,40,41,42]. MOF-derived electrocatalysts, with their adjustable composition, expansive specific surface area, controlled shape, high porosity, and thermal stability, have demonstrated promising potential in rivaling their noble metal counterparts [25,26,27,28,43,44,45]. Moreover, the active sites typically undergo dynamic changes during the electrolytic process, suggesting that the actual active sites during electrocatalysis may not be the components that were characterized in the original structure [46,47,48]. In the context of the aforementioned MOF-derived nanostructures, the conductivity can be improved by applying an additional conductor to the MOF precursors. This supplementary one-dimensional conductor serves to prevent the aggregation of morphological regular MOFs. For example, Hu [49] fabricated N, P co-doped porous carbon capsules expressing self-phosphatized metal phosphides, with a MOF coating serving as an armoring layer for facilitating morphology inheritance from bio-templates and providing metal sources for generating extra porosity and electrochemically active sites. Thus, the P-rich phospholipids and N-rich proteins from the plasma membrane enable carbon matrix doping and further yield metal phosphides, ultimately leading to the superb performance of catalyzing reversible oxygen conversion and zinc–air batteries. Yan [50] grew CoS/Fe_3_S_4_ nanoparticles on S, N co-doped carbon plate arrays, where the hydrophobic–aerophilic surface can repel water molecules to create abundant solid–liquid–gas three-phase reaction interfaces, as well as exposing Fe-sites, which consequently promote the diffusion of reactive molecules/ions across the interface and oxygen adsorption. Pan [51] synthesized a CoNiPt alloy embedded in N-doped porous carbon with a nanoflower (NF)-like hierarchy structure by pyrolyzing Hofmann-type metal–organic frameworks. Because of the synergistic interaction between oxygen defects and pyrrolic/graphitic N species, the adsorption energy of the intermediate species in the ORR process was optimized and catalytic activity was greatly enhanced.

Considering the aforementioned factors, we developed a simple method to synthesize a ZIF-L/carbon nanofiber composite material. This material leverages the high conductivity of carbon fibers and the high catalytic activity of ZIF-L derivatives. As a result, the ORR and OER performances of these structures are remarkable, matching the benchmarks set by 20 *wt*% Pt/C and RuO_2_, but with the added benefit of superior long-term stability. This underscores the potential of these MOF-derived nanostructures in the field of electrocatalysis.

Moreover, the excellent electrochemical activities of the catalyst are clearly demonstrated in rechargeable zinc–air batteries, which display exceptional energy conversion efficiency and excellent charge and discharge stability. However, there is a noticeable performance decline during the early stage of the catalytic cycle. Future research will focus on the reasons for this decline in cyclability. Lastly, the transformation of catalytic sites during electrolysis is meticulously examined through detailed control experiments and post-catalytic characterizations. This provides valuable insights into the origin of the activity and the dynamic evolution of the catalyst during the electrolysis process. This understanding is crucial for the further development and optimization of these electrocatalysts.

## 2. Results and Discussion

### 2.1. Material Characterization

**Figure 1:**
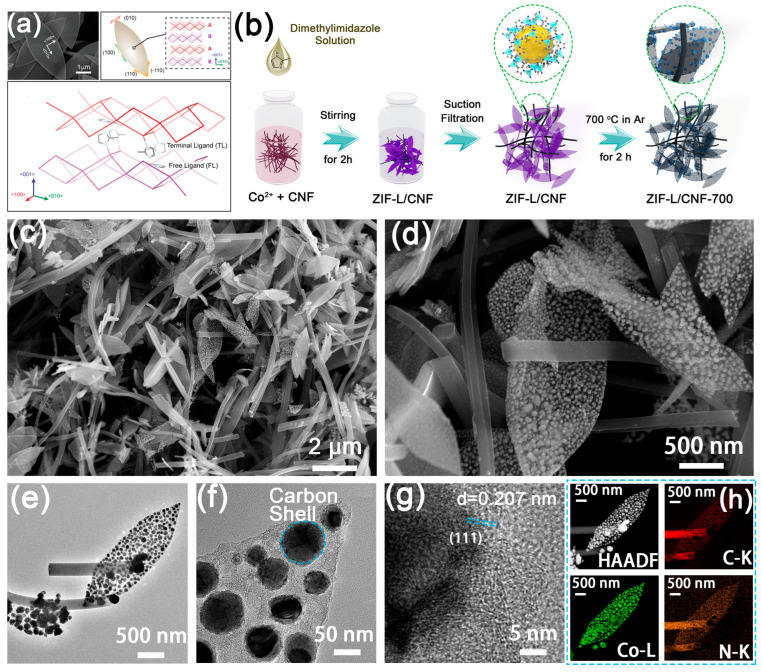
(**a**) Growth mechanism of ZIF-L [52,53]. (**b**) The fabrication process of ZIF/CNF-700. (**c**–**g**) Morphological and microstructural characterizations of ZIF-L/CNF-700. (**c**,**d**) SEM and (**e**,**f**) TEM images of ZIF-L/CNF-700. (**g**) HR-TEM image on a conglomerated particle of Co. (**h**) EDX-mapping images of ZIF-L/CNF-700.

The leaf-like hierarchical carbon structure, which is embedded with Co nanoparticles, was synthesized by pyrolyzing ZIF-L/CNF in argon at varying temperatures (refer to Figure 1b). This structure is referred to as ZIF-L/CNF-X, where ‘X’ represents the annealing temperature. Notably, the leaf-like structure and stable porous state of ZIF-L allow for the preservation of their hierarchical morphology throughout the pyrolysis process.

Since it was synthesized in an aqueous solution, the crystal structure could easily grow along the <100> and <010> crystal planes to form leaf-shaped ZIF-L [52,53,54,55,56,57,58,59]. After Co and dimethylimidazole were combined in a stirring aqueous solution, the leaf-shaped ZIF-L and CNF were stacked on each other. Excess ions and impurities were then removed through suction filtration, followed by calcination at a high temperature under an argon atmosphere. Scanning electron microscopy (SEM) images (Figure 1c,d) of ZIF-L/CNF-700 revealed that high-temperature carbonization transformed the regular leaf-shaped ZIF-L into a porous carbon framework with a similar morphology. The Co^2+^ in ZIF-L was converted into corresponding metal particles, which were evenly dispersed in the carbon framework. The carbon fiber, interspersed in the leaf-shaped carbon frame, maintains a one-dimensional shape, playing a conductive and supporting role.

After thermal treatments at 600, 700, 800, and 900 °C, the leaf-shaped structures were well preserved when compared with the original ZIF-L/CNF (Figure S1), and the carbonized Co particles clustering along the carbon frame exhibited slightly shrunken and rougher surfaces. Co particles were visible on samples carbonized from 600 to 900 °C, with a growing particle diameter from ZIF-L/CNF-600 to ZIF-L/CNF-900, indicative of aggregated cobalt nanoparticles and Ostwald ripening caused by high-temperature annealing. Statistical analyses (Figures S2–S5) on the particle size distribution show that the average size of conglomerated particles inside the ZIF-L structure upon thermal annealing increases from 49 ± 20, 93 ± 21 nm, and 118 ± 50 to 139 ± 23 nm for ZIF-L/CNF-600, ZIF-L/CNF-700, ZIF-L/CNF-800, and ZIF-L/CNF-900, respectively.

The high-resolution TEM characterization of ZIF-L/CNF-700’s nanostructure (Figure 1e–g) reveals a core–shell structure, with a cobalt-based core and ZIF-L-based scaffolds forming the shell. The metal particles have an interplanar spacing of 0.207 nm, aligning with the (111) crystal plane of Co. This is further confirmed by the selected-area electron diffraction (SAED) patterns (Figure S6), which indicate polycrystalline cobalt phases. The interior diffraction ring of the SAED image is attributed to the amorphous carbon shell. Energy-dispersive X-ray (EDX) mapping images (Figure 1h) taken under a high-angle annular dark-field scanning transmission electron microscope reveal that Co elements are located exclusively on the aggregated particles, while C and N are primarily enriched in the leaf’s carbon shell. The doping of N into the carbon matrix is considered beneficial for enhancing catalytic activities, as per previous reports. Furthermore, the actual elemental content in all samples was examined using EDX and corroborated by X-ray photoelectron spectroscopy (XPS). These analyses consistently reveal the differences and regularities in the contents of Co, C, and N elements, as shown in Table S1.

**Figure 2:**
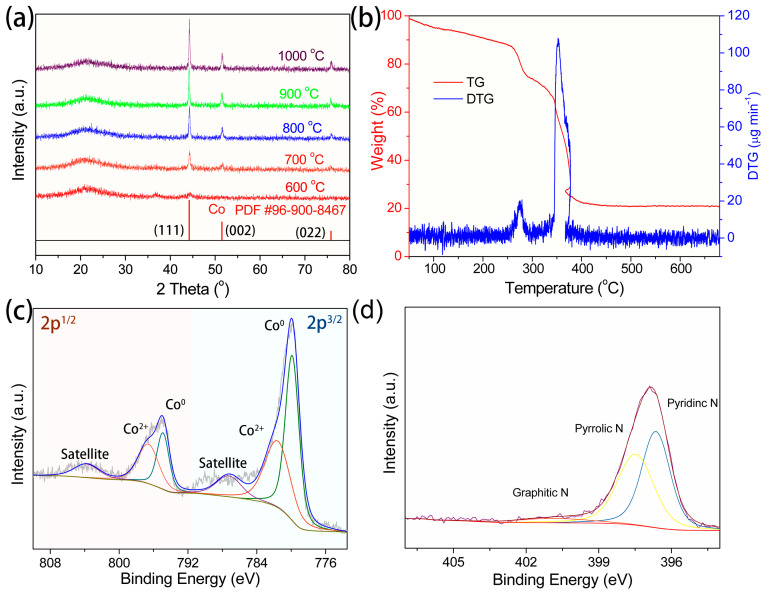
Spectroscopy analyses of ZIF-L/CNF. (**a**) XRD patterns of ZIF/CNF with different annealing temperature (**b**) the TGA of ZIF-L/CNF in Ar atmosphere and (**c**) High-resolution XPS spectra of Co 2p and (**d**) N 1s spectra of ZIF-L/CNF-700.

The agglomerated particles within the carbon nano-leaves were corroborated by X-ray diffraction (XRD) analysis. As shown in Figure 2a, the XRD pattern of metallic cobalt (PDF #96-900-8467) in ZIF-L/CNF-700 at the comparatively lower temperatures of 600 and 700 °C is subdued, particularly at 600 °C, signifying its low crystallinity. With the rise in calcination temperature, the peak of metallic Co ascends, indicating a steady enhancement in crystallinity during the annealing process. This is substantiated by three distinct peaks at 44.17°, 51.47°, and 75.77°, corresponding to the (111), (002), and (022) planes, respectively. All the microscopic and spectroscopic evidence conclusively demonstrates the successful formation of N-doped nano-leaves of the carbon frame, encapsulating metallic cobalt clusters. The mass percentage and thermal stability of the carbon fiber composites were further ascertained by thermal gravimetric analysis (TGA) (Figure 2b) in an Ar atmosphere. Owing to the loss of adsorbed water, the weight of the composites gradually decreased from 50 to 250 °C. During this phase, the material shed 10% of its weight, suggesting that despite the extended drying during the preparation process, the surface still retained a significant amount of water due to its high specific surface area. Between 250 and 400 °C, the material underwent a sharp decline in quality, from 90% to 30%, corresponding to the decomposition and dehydration of ZIF-L. Post 400 °C, the material entered a long-term stable phase. In this phase, the generated carbon framework ceased to decompose abruptly, while growth of the metal nanoclusters persisted. Raman characterization (Figure S7) reveals two distinct peaks centered at 1340 cm^−1^ and 1581 cm^−1^, corresponding to the G and D bands of carbon. This indicates the presence of graphitization and defects within the carbon framework. The ID/IG ratio, which decreases from 1.27 for ZIF-L/CNF-600 to 1.14 for ZIF-L/CNF-900 with the increasing annealing temperature, suggests enhanced carbonization, more pronounced metal reduction and the loss of N dopants.

The catalyst’s pore structure is crucial for its performance. BET analysis of ZIF-L/CNF-700 was conducted to better understand the composite’s pore structure (Figure S8). The analysis revealed a type-IV isotherm, suggesting the presence of abundant mesopores within the structure of the nano-leaves. The specific surface area and average pore size were found to be 325 m^2^ g^−1^ and 9.5 nm, respectively, highlighting the importance of preserving the morphology of the nano-leaves for improved porosity and surface area.

X-ray photoelectron spectroscopy (XPS) was used to analyze the surface elemental composition and chemical states of ZIF-L/CNF-700. The survey spectrum (Figure S9) shows the presence of Co, C, N, and O. The oxygen may be due to absorbed oxygen species and surface oxidation from air exposure. The high-resolution XPS Co 2p spectrum of ZIF-L/CNF-700 (Figure 2c) shows binding energy peaks at 779.4 and 793.6 eV, attributed to the 2p^3/2^ and 2p^1/2^ spin–orbitals of Co in the Co nanoparticle phase, derived from the Co^2+^ ion in ZIF-L during annealing. Peaks at 782.3 and 796.9 eV correspond to the 2p^3/2^ and 2p^1/2^ states of Co^2+^, resulting from surface oxidation upon air exposure. Satellite peaks at 787.3 and 803.8 eV are likely due to the shakeup excitation of high-spin Co^2+^ ions [60,61]. The C1s spectrum analysis (Figure S10a) confirms the successful doping of N into the carbon, with the coexistence of C-C (284.1 eV), C-N (285.2 eV), and C-O (289.1 eV). The N 1s spectrum can be deconvoluted into subpeaks of pyridinic-N at 397.8 eV, pyrrolic N at 398.7 eV, and graphitic-N at 400.4 eV (Figure 2d). Similarly, it can be seen from the O 1s spectrum that the M-O peak appears at 529.1 eV, and the peak at 531.0 eV is OHad. The M-O peak is weak, indicating that the catalyst surface is partially oxidized [62].

### 2.2. Electrocatalytic Performance

**Figure 3:**
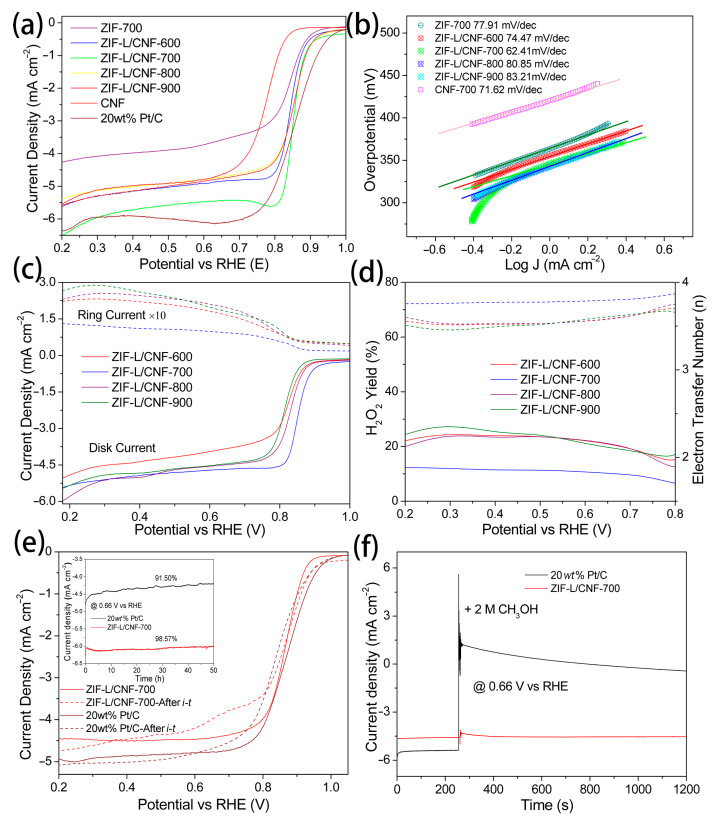
ORR performances of ZIF-L/CNF-700 vs. 20 *wt*% Pt/C. (**a**) RDE LSV curves with a scan rate of 5 mV s^−1^. (**b**) The corresponding Tafel slopes of (**a**). (**c**) RRDE LSV curves with ring currents magnified by ten times. (**d**) Plots of electron transfer number and peroxide yield. (**e**) LSV curves obtained before and after the *i*–*t* tests for ZIF-L/CNF-700 (inset: Chronoamperometric *i*–*t*). (**f**) The CH_3_OH crossover reaction in chronoamperometric ORR test at 0.66 V vs RHE.

The oxygen reduction reaction (ORR) performance of the synthesized ZIF-L/CNF-700 was evaluated using a rotating disk electrode (RDE) electrolytic system in an O_2_-saturated 0.1 m KOH electrolyte. The cyclic voltammetry (CV) curves, depicted in Figure S11, were recorded at a scan rate of 100 mV s^−1^ within a voltage range of 1.24 to 0.04 V (vs. RHE). Contrary to the results in N_2_, ZIF-L/CNF-700 demonstrates an ORR activity with a significant cathodic peak at 0.805 V in the presence of O_2_, while the redox peak at 1.09 V can be ascribed to the redox of the material itself. More quantitatively, the ORR polarization curves of ZIF-L/CNF-700 (Figure 3a) exhibit a half-wave potential (E_1/2_) of 0.852 V and a diffusion-limited current density of 5.73 mA cm^−2^ (@0.40 V, 1600 rpm). In contrast, ZIF-L-700 without the integration of CNF displays a half-wave potential (E_1/2_) of 0.844 V and a diffusion-limited current density of 3.96 mA cm^−2^. By analyzing the differences, it can be observed that the E_1/2_ is similar, but the diffusion-limited current varies. This aligns with the roles in the composite: ZIF-L-700 functions as the catalytically active component, while CNF acts as the conductive and structural frame component. For comparison, the benchmark 20 *wt*% Pt/C exhibits an E_1/2_ of 0.860 V and a diffusion-limited current density of 5.93 mA cm^−2^, which is only 5 mV and 0.2 mA cm^−2^ greater than that of ZIF-L/CNF-700. Consequently, the lowest Tafel slope of 62.41 mV dec^−1^ was observed for ZIF-L/CNF-700, which shows that for every tenfold increase in current, the required overpotential increase is only 64mV, indicating highest ORR kinetics (Figure 3b). This trend in ORR activity is further substantiated by the measurements of Tafel slopes, displaying values of 74.47, 80.85, 83.85 mV dec^−1^ for ZIF-L/CNF-600, ZIF-L/CNF-800, and ZIF-L/CNF-900, respectively (Figure 3c).

The electron transfer number (n) and peroxide (HO_2_^−^) generation during ORRs were further examined by the rotating ring disk electrode (RRDE) by maintaining the potential of the ring disk at 1.55 V versus RHE with a current collection efficiency of 0.37. As illustrated in Figure 3d, in contrast, ZIF-L/CNF-700 exhibits a significantly lower ring current, while the disk current is comparable to other samples. Correspondingly, the average H_2_O_2_ yield for ZIF-L/CNF-600, ZIF-L/CNF-700, ZIF-L/CNF-800, and ZIF-L/CNF-900 is 20.25%, 10.92%, 23.07%, and 24.50%, respectively. Additionally, the average electron transfer numbers are 3.52, 3.78, 3.58, and 3.50, respectively. The high n value and low H_2_O_2_ yield observed for ZIF-L/CNF-700 confirm a 4e^−^ participated ORR process, further substantiated by the K-L plots depicted in Figure S12. The electrocatalytic stability of ZIF-L/CNF-700 was probed by the extended chronoamperometric i–t test at 0.66 V versus RHE (Figure 3e). The current density of the latter remains at 98.55% after 50 h. Moreover, the polarization curves before and after the i–t test exhibit negligible change in both half-wave potential and diffusion-limited current density for ZIF-L/CNF-700, endorsing its excellent electrochemical endurance. Furthermore, compared to 20 *wt*% Pt/C, the ZIF-L/CNF-700 catalyst also demonstrated superior tolerance to foreign contaminants such as CH_3_OH (Figure 3f), typically used to study the catalyst poisoning by organic agents.

In addition to ORR, ZIF-L/CNF-700 also showcases remarkable OER performance (Figure S13). Among all the ZIF-L/CNF samples, the catalyst annealed at 700 °C delivered the highest OER activity (Figure S13a) by exhibiting an onset overpotential (η_onset_) of 300 mV and an overpotential of 340 mV to reach the anodic current density of 10 mA cm^−2^ (η_10_). For comparison, the η_onset_ and η_10_ for the benchmark RuO_2_ are 240 and 320 mV, respectively. Similar to the ORR scenario, the Tafel slopes (Figure S13b) of all ZIF-L/CNF samples align with that of RuO_2_ (53.77 mV dec^−1^) with the lowest value of 68.68 mV dec^−1^ observed for ZIF-L/CNF-700, suggesting superior OER kinetics. Excellent electrolytic OER stability was also observed during the 10 h chronoamperometric i–t test held at a fixed voltage of 1.57 V, showing a stabilized current of ~10 mA cm^−2^ throughout the testing period and nearly identical polarization curves before and after the extended test (Figure S13c). In general, the above electrochemical tests collectively testify ZIF-L/CNF-700 as a promising bifunctional ORR and OER catalyst comparable to 20 *wt*% Pt/C and RuO_2_ in catalyzing reversible oxygen conversion. The electrochemically active surface area (ECSA) (Figure S14) of ZIF-L/CNF-X was estimated by calculating the double-layer capacitance from cyclic voltammetry (CV) curves at different scan rates. A linear correlation can be observed when the current density is plotted against the scan rate for all samples. ZIF-L/CNF-700 possesses the largest C_dl_ (26.9 mF cm^−2^) amongst all ZIF-L/CNF-X samples, indicative of the highest surface area and exposure of active sites. 

**Figure 4:**
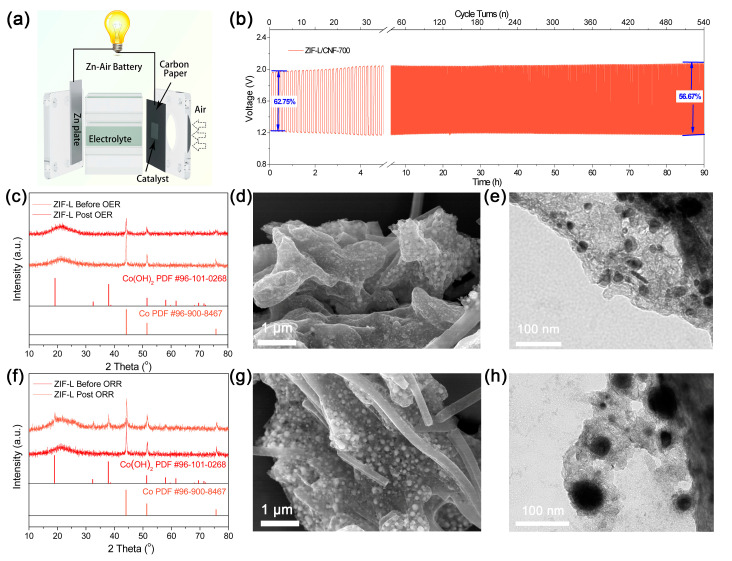
Performance of the aqueous ZABs using the bifunctional ZIF-L/CNF-700 as the air cathode catalyst. (**a**) Device diagram of zinc air battery. (**b**) Long-term galvanostatic cycling test showing the decreased catalytic activity early in the catalytic cycle. Post-OER and post-ORR characterizations of ZIF-L/CNF-700. (**c**) XRD patterns of ZIF/CNF-700 before and after OER; (**d**) SEM and (**e**) TEM images of the post-OER catalyst retaining the leaf-like morphology but significantly smaller particles; (**f**) XRD patterns of ZIF/CNF-700 before and after ORR; (**g**) SEM and (**h**) TEM images of the post-ORR catalyst retaining the leaf-like morphology but slightly puffy particles.

Given the exceptional ORR and OER performance of ZIF-L/CNF-700, rechargeable Zn–air batteries (ZABs) were demonstrated using this bifunctional catalyst in ambient atmosphere without the need for external oxygen purging (Figure 4a). Notably, both the charge and discharge curves of ZIF-L/CNF-700 surpass the benchmark of mixed 20 *wt*% Pt/C+RuO_2_ (Figure S15a), aligning with its superior ORR and OER characteristics. Subsequent open circuit voltage tests (Figure S15b) revealed that the open circuit voltage of batteries assembled from this material can reach 1.40 V, while the corresponding 20 *wt*% Pt/C+RuO_2_ has an open circuit voltage of 1.42 V, mirroring the difference in ORR and OER onset potential between the two catalysts. A maximum power density of 146 mW cm^−2^ was achieved by ZIF-L/CNF-700, slightly exceeding that of 20 *wt*% Pt/C+RuO_2_ (Figure S15c). Similarly, the rate tests displayed in Figure 4d show a lower voltage output by ZIF-L/CNF-700 when the discharge rate is less than 5 mA, but it significantly outperforms the 20 *wt*% Pt/C + RuO_2_ benchmark beyond that, owing to the lower ORR Tafel slope of ZIF-L/CNF-700 observed in Figure 3a and b. The total discharge capacity normalized to Zn consumption is measured at 781 mA h g_Zn_^−1^ at 20 mA, which is 95.4% of the theoretical specific capacity of 819 mA h g_Zn_^−1^ (Figure S15e). The long-term cycling stability of ZIF-L/CNF-700 was evaluated using a galvanostatic cycling test at 10 mA cm^−2^, enduring for over 90 h with a retained energy efficiency of 56.6% at the end of the test (Figure 4b). Interestingly, the cycle efficiency was relatively high at the beginning, reaching 62.75%, but after 90 h of cycling, it dropped to 56.67%. The material’s performance degrades most severely within the first 4 h, potentially due to the catalyst undergoing redox during the reaction. The subsequent CV test (Figure S15f) also confirmed the redox reaction of the catalyst under the applied voltage range, such as the redox peak pair at 1.2 V and 1.3 V, indicating that the catalyst is not static during the catalytic reaction, but evolves over time, which is the actual reason for the decline in ZAB performance. While previous ORR and OER stability tests indicated good catalyst stability, the catalyst significantly decreases in the ZAB cycle, suggesting some catalyst deactivation. Further characterization of the material after the catalytic reaction could help identify the cause of this performance decline.

Given that both the cobalt cluster and nitrogen-doped carbon framework can readily undergo oxidative transformation under alkaline conditions at the potentials applied during ORR and OER, it is crucial to thoroughly characterize both post-ORR and post-OER samples to examine the catalyst composition’s evolution. After a prolonged OER stability test in 1.0 M KOH for 5 h, the original XRD spectrum’s diffraction peaks (Figure 4c) were altered and replaced with low-intensity peaks of low crystallinity cobalt particles. While the cubic morphology of ZIF-L/CNF-700 remained (Figure 4d), TEM images of the post-OER catalyst revealed a reduction in the size of the original cobalt nanoparticles within the leaf-like carbon shell, except for significantly reduced metal particles with a smaller diameter (Figure 4e). To trace the whereabouts of metallic Co and the dynamic changes in carbon frames, a post-OER XPS characterization of Co, C, and N was conducted. As shown in Figure S17a, the small peaks at 778 eV and 794 eV corresponding to metallic Co 2p_3/2_ and 2p_1/2_ decreased significantly after OER. Correspondingly, the peaks attributed to Co^3+^ and Co^2+^ increased significantly. This is consistent with the XRD data, indicating that the metal particles are partially oxidized during the OER process, with the metal particles being converted into metal ions without forming well-crystallized metal hydroxides. Additionally, the C and N spectrum in Figure S18a–c shows a significant improvement in the intensity of the C-O N-O species and M-O, confirming that not only the metal will face oxidation, but the C and N elements of the carbon framework will also be oxidized accordingly.

Conversely, the catalyst after 5 h of ORR testing in 0.1 M KOH exhibits a completely different state. The diffraction peaks of Co nanoparticles in the original XRD spectrum (Figure 4f) slightly decrease, while low-intensity peaks of corresponding hydroxide appear, suggesting the partial conversion of metal clusters into metal hydroxides. Subsequent SEM characterization (Figure 4g) reveals that the carbon frame remains intact, while the nanoparticles become slightly fluffy. To delve deeper, TEM was employed (Figure 4h). Notably, the carbon framework remains intact, while lamellar-like structures form around the metal particles. Combined with XRD data, it is speculated that the central part still comprises metal nanoparticles, while the surface-surrounded flakes are the in situ converted cobalt hydroxide.

Incidentally, the post-ORR XPS characterization of Co, C, and N was also conducted. In terms of the Co 2p spectrum (Figure S17b), it was found that the peaks of metallic Co have disappeared, replaced by the peaks of metal hydroxide. This is somewhat inconsistent with the XRD and TEM data, which suggest that a large amount of metallic Co remains. This phenomenon can be attributed to the surface inspection characteristics of the XPS test, while the metallic Co nanoparticles are wrapped inside the cobalt hydroxide. The subsequent XPS peaks of C (Figure S19a), N (Figure S19b), and O (Figure S19c) also show that the carbon framework was significantly oxidized. Judging from the ratio of oxidation peaks, the oxidation degree of the carbon framework is much higher than that after ORRs, intimating that OERs are more destructive to carbon frames than ORRs, which also illustrates the dynamic changes in the catalyst during the catalytic process [63,64,65].

Finally, as a result of the above in-depth investigation and post-electrolytic analyses, it can be naturally derived that one of the reasons for the decrease in ZAB efficiency is the transformation of the active site. The oxidation of the carbon framework can be attributed to the oxidation caused by high voltage. However, the reason for the transformation of metal nanoparticles after OERs and ORRs remains to be discussed. Here, we surmise that the local pH fluctuation at the catalyst/electrolyte interface caused by quick OH^−^ consumption and production during OERs/ORRs might play a crucial role in the transformation of Co nanoparticles. Since the OER consumes OH^−^, the local pH of the catalyst surface decreases, and Co particles will be dissolved in the electrolyte along with the oxidation process, therefore reducing particle size. Conversely, for ORR, which generates OH^−^, the local pH of the catalyst surface increases; therefore, the generated Co(OH)_2_ will tightly cover the surface of Co particles. 

## 3. Conclusions

In this study, leaf-like N-doped carbon-frame embedding nanoparticles were fabricated by pyrolyzing the composite, ZIF-L/CNF, with CNF as a conductive and framing material and ZIF-L derivatives as catalytically active sites. As a result, the optimized catalyst of ZIF-L/CNF-700 achieved remarkable catalytic activities in both ORRs (*E*_1/2_ = 0.852 V) and OERs (η_10_ = 1.56 V), comparable to those of the benchmark 20 *wt*% Pt/C and RuO_2_, with even superior kinetics reflected by the lowered Tafel slopes. As a result of the much-improved electrochemical activities, high-performance rechargeable ZABs were demonstrated with superior power conversion efficiency comparable to that of the state-of-the-art 20 *wt*% Pt/C and RuO_2_ catalysts. Aiming to solve the problem of reduced cycle performance in the ZAB cycle process, further in-depth investigations on post-electrolytic composition revealed that both carbon frameworks and Co nanoparticles are partially oxidized during both OERs and ORRs. Due to the different local pH of the catalyst surface, the oxidized metal nanoparticles present distinct microstructures, with reduced-size Co particles as the product after the OER, and Co particles wrapped with Co(OH)_2_ in the outer layer as the ORR product.

## Data Availability

Data is contained within the article and Supplementary Materials.

## Supplementary Materials

The following supporting information can be downloaded at: https://www.mdpi.com/article/10.3390/molecules29184494/s1. Experimental details: Figure S1–S19 and Table S1.
