# Supplementary material for: Unveiling the Dynamic Evolution of Catalytic Sites in N-Doped Leaf-like Carbon Frames Embedded with Co Particles for Rechargeable Zn–Air Batteries
Source: Molecules. 2024 Sep 22;29(18):4494. doi: 10.3390/molecules29184494 (PMC11434714; doi:10.3390/molecules29184494)
Supplement: Supplementary file 1 [file molecules-29-04494-s001.zip › molecules-3206187-supplementary.pdf]

Unveiling the Dynamic Evolution of Catalytic Sites in N doped Leaf-like Carbon Frame Embedding Co Particles toward Rechargeable Zn-air Batteries

Yuebin Lian, [1]* **Weilong Xu,**[1] **Xiaojiao Du,**[1] **Yannan Zhang,**[1] **Weibai Bian,**[1 **Yuan Liu,**[3] **Jin Xiao,**[1] **Likun Xiong,**[4] **Jirong Bai** [2]*

1 Dr. Y. B. Lian, Dr. W. L. Xu, Dr. X. J. Du, Dr Y. N. Zhang, Dr. Y. L, Hong, Dr. J. Xiao, School of Optoelectronic Engineering, Changzhou Institute of Technology, Changzhou 213032, P. R. China.

2 Dr. J. R. Bai Research Center of Secondary Resources and Environment, School of Chemical Engineering and Materials, Changzhou Institute of Technology, Changzhou 213032, P. R. China.

3 Dr Y. Liu, School of Chemistry and Chemical Engineering, Henan University of Technology, Zhengzhou 450001, P. R. China;

4 Dr. L. K. Xiong, School of Chemistry and Environmental Engineering, Shanghai Institute of Technology, Shanghai, 201418, P.R. China.

Experimental details

Materials

Chemicals: All chemicals, $Co(NO_3)_2{\cdot}6H_2O$ (98+%, Adamas, Switzerland), C_2H_5OH (Enox, China), Methylimidazole (2-MeIM) was obtained from Meryer (Shanghai) Chemical Technology Co., Ltd., N,N-dimethylformamide (DMF) (≥99.5%, Greagent, China), and polyacrylonitrile (PAN) (Mw 200000, Polysciences, USA) were used as obtained.

Preparations

Preparation of ZIF-L/CNF: Initially, 0.5 g of PAN was introduced into 5 mL of DMF. This mixture was agitated at 50 °C until a transparent solution was obtained. The solution was then transferred into a plastic syringe equipped with a stainless-steel needle. The electrospinning process was conducted at 17 kV, with the needle and collector positioned 10 cm apart and a flow rate of 13 μL min^{-1}.

Subsequently, the resulting film was dried at 60 °C overnight. The dried film was then heated at a rate of 2 °C min^{-1} up to 300 °C, and maintained at this temperature for 2 hours in an air atmosphere. Following this, the peroxidized film was carbonized at 1000 °C at a ramping rate of 5 °C min^{-1}, and held at this temperature for 2 hours in a nitrogen atmosphere within a tube furnace.

The carbon nanofiber (CNFs) were then sonicated in 80 mL of a nitric acid and sulfuric acid mixture (volume ratio 1:3) for 1 hour, followed by refluxing at 80 °C for an additional 4 hours. After cooling, the CNFs were rinsed with distilled water until the pH of the solution reached 7. Finally, the CNFs were collected via filtration and dried for subsequent use.

A water mixture of dimethylimidazole and CNF (mass ratio 4:1) was thoroughly mixed, after which a Co^{2+} solution was added dropwise. After stirring continuously for 4 hours, the purple-black precipitate was filtered, washed, and dried for future use.

Preparation of ZIF-L/CNF-X:100 mg ZIF-L/CNF powder was annealed at 700 C for 2 hours in a tube furnace with a heating rate of 5 °C min^{-1} under Ar (100 mL min^{-1}) atmosphere After annealing, the carbonized ZIF-L/CNF was denoted as ZIF-L/CNF-700.

ORR tests

Electrochemical assessments were conducted using a CHI 760E electrochemical station within a standard three-electrode system with 0.1 M KOH. The working electrode was a glass carbon rotating disk electrode (RDE) with a diameter of 5 mm and an area of 0.196 cm^2. A saturated KCl Ag/AgCl electrode and a carbon rod served as the reference and counter electrodes, respectively. All potentials were adjusted to the Reversible Hydrogen Electrode (RHE) scale using the equation E (vs RHE) = E(vs Hg/HgO) + 0.059 pH + 0.098 V.A homogeneous ink was prepared by dispersing 4 mg of catalysts and 1 mg of Ketjen Black (KB) in a solution containing 800 μL of water, 200 μL of ethanol, and 50 μL of 5 wt% Nafion, followed by 30 minutes of sonication. This ink was then applied to the glass carbon (GC) electrode, with 20 μL of the catalyst ink used for the Oxygen Reduction Reaction (ORR). Before the Linear Sweep Voltammetry (LSV) tests, the reaction cell was purged with O_2 for 20 minutes and subjected to 100 Cyclic Voltammetry (CV) scans. The CV scans were performed at a rate of 100 mV/s within a potential window of 1.07 V to 0.07 V (vs RHE). LSV curves were recorded at a scan rate of 10 mV/s, with the RDE rotation rate varying from 400 to 2500 rpm. The number of electrons transferred (n) and the yield of peroxide in the ORR process were calculated using the Koutecky-Levich (K-L) equation.

$$J^{-1} = J_k^{-1} + \left(B\omega^{1/2}\right)^{-1}$$

$$B = 0.62nF(D_0)^{2/3}\nu^{-1/6}C_0$$

$$J_k = nFkC_0$$

where J is the measured current density during ORR, J_k is the kinetic current density, ω is the electrode rotating angular velocity (ω = 2πN, N is the linear rotation speed), B is the slope of K-L plots, n represents the electron transfer number per oxygen molecule, F is the Faraday constant (F = 96485 C mol^{-1}), D_0 is the diffusion coefficient of O_2 in 0.1 M KOH (1.9×10^{-5} cm^2 s^{-1}), ν is the kinetic viscosity (0.01 cm^2 s^{-1}), C_0 is the bulk concentration of O_2 (1.2×10^{-3} mol L^{-1}).

Cyclic voltammetry (CV) curves were utilized to investigate the electrochemical capacitance of the catalysts. For the RRDE measurements, the ring potential was set to constant at 1.55 V vs RHE.

The peroxide yield (HO_2^-%) and electron transfer number (n) were determined by the followed equations:

$$n = \frac{4 \times i_d}{i_d + \frac{i_r}{N}}$$

$$HO_2^- \% = \frac{200 \times \frac{i_r}{N}}{i_d + \frac{i_r}{N}}$$

Aqueous ZAB Assembly

The cathode was prepared by pipetting the catalyst ink onto a carbon paper (CP) with a loading amount of 1 mg cm^{-2} and dried at 60 °C for 2 h. A polished Zn plate (300 μm in thickness) was applied as the anode, and 6 M KOH with 0.2 M $Zn(Ac)_2$ was used as the electrolyte for the homemade rechargeable ZAB batteries. Cell performances were measured at room temperature on the CHI 760E electrochemical workstation and LANHE battery testing system.

Battery capacity calculation

$$\boldsymbol{Theoretical\ capacity} = \frac{\boldsymbol{1g \times 96485C/mol \times 2}}{\boldsymbol{65.38\ g/mol}} = \boldsymbol{2951.5C/g = 819mAh/g}$$

$$\boldsymbol{Actual\ capacity} = \frac{\boldsymbol{Rated\ current \times Time}}{\boldsymbol{Mass\ of\ zinc\ consumed}}$$

Characterizations

The product's crystalline phase was analyzed using a Bruker D8 Advance X-ray diffractometer for powder XRD measurements, utilizing Cu-Ka radiation at a scanning rate of 0.1 s^{-1}. The optimal calcination temperature for achieving the best morphology and structures was determined through TGA, using an EXSTAR 7300 from SII Nano Technology. The characteristic vibrational modes of the synthesized materials were identified through Raman spectrum analysis, conducted with a Horiba HR Evolution, using a laser excitation at 633 nm and a sweep from 200 to 2500 cm^{-1}. The specific surface area of the catalysts was determined using Brunauer-Emmett-Teller (BET) analysis, performed with a Micromeritics Tristar II 3020. X-ray photoelectron spectroscopy (XPS), conducted with a Thermo Fisher Escalab 250Xi using Mg Ka X-ray as the excitation source, was used to analyze the surface elemental states of the samples. The samples' morphologies were examined using a Hitachi SU8010 scanning electron microanalyzer, operating at an accelerating voltage of 10 kV. The samples' microstructure was characterized using a FEI TECNAI G20 field-emission TEM, operating at 200 kV.

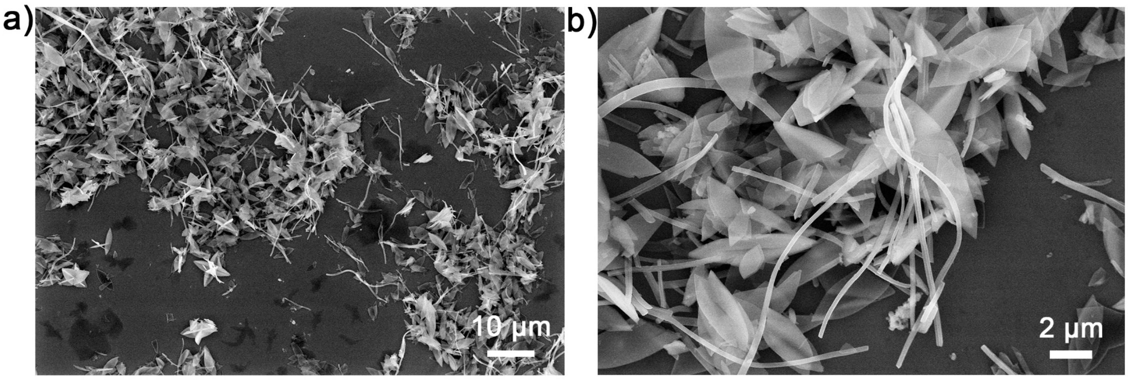

Figure S1. The a) and b) SEM image with different magnifications of ZIF/CNF.

Table S1 Element content of ZIF/CNF-700 quantified by different methods

Content	Co (atom%)	N (atom%)	C (atom%)
XPS	12.05	1.28	86.67
EDX	11.32	1.38	87.29

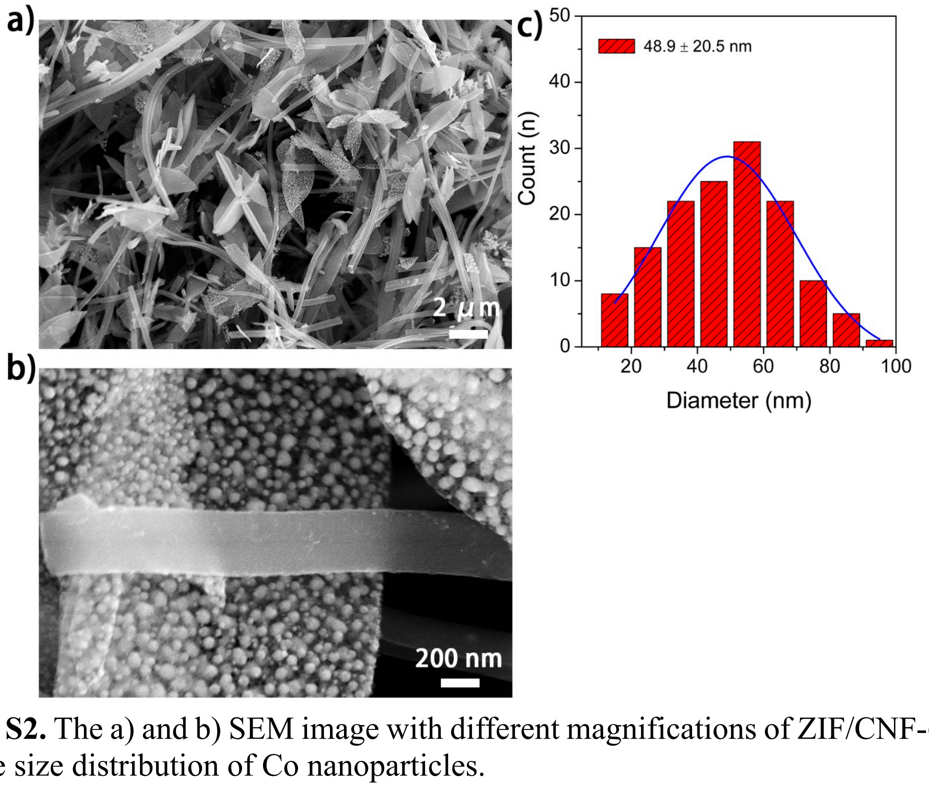

Figure S2. The a) and b) SEM image with different magnifications of ZIF/CNF-600; c) Particle size distribution of Co nanoparticles.

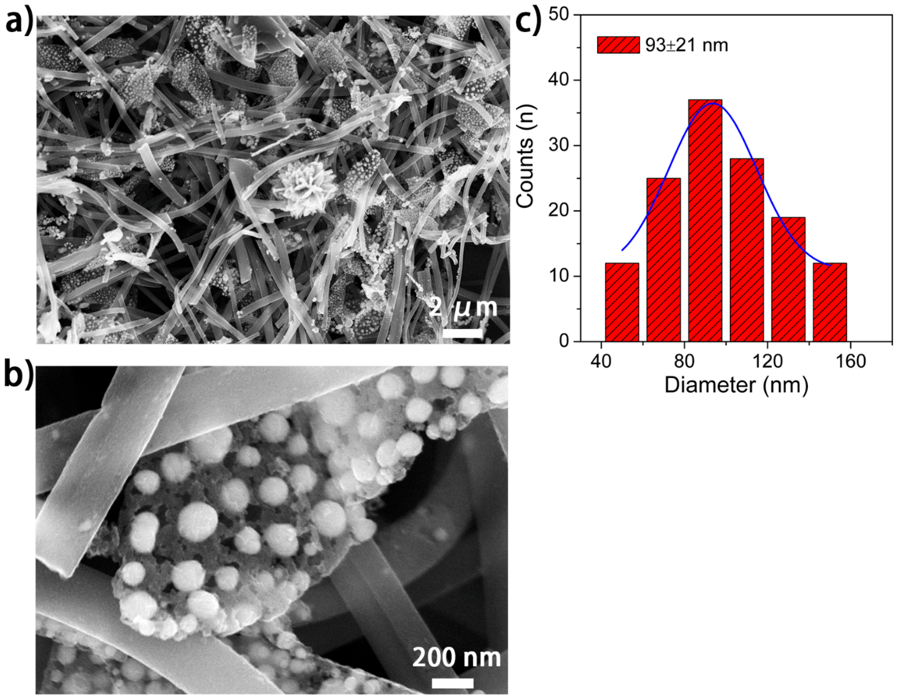

Figure S3. The a) and b) SEM image with different magnifications of ZIF/CNF-700; c) Particle size distribution of Co nanoparticles.

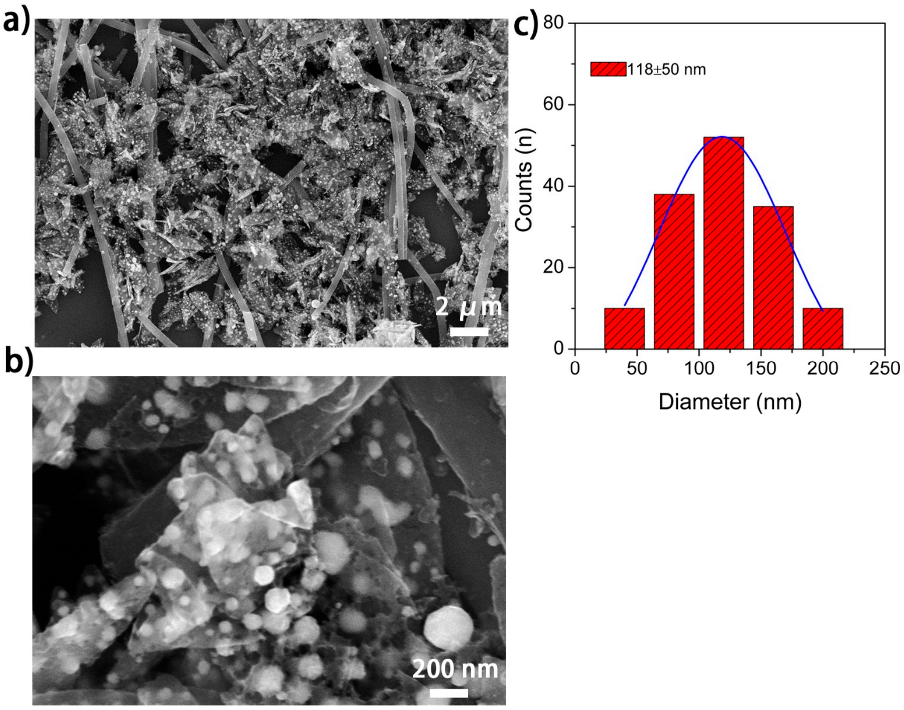

Figure S4. The a) and b) SEM image with different magnifications of ZIF/CNF-800; c) Particle size distribution of Co nanoparticles.

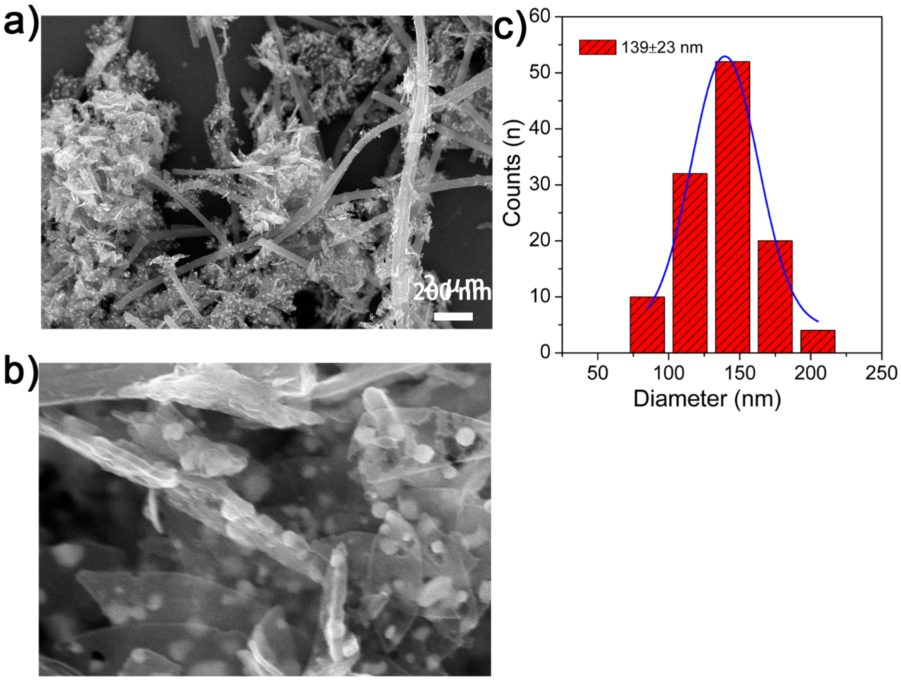

Figure S5. The a) and b) SEM image with different magnifications of ZIF/CNF-900; c) Particle size distribution of Co nanoparticles.

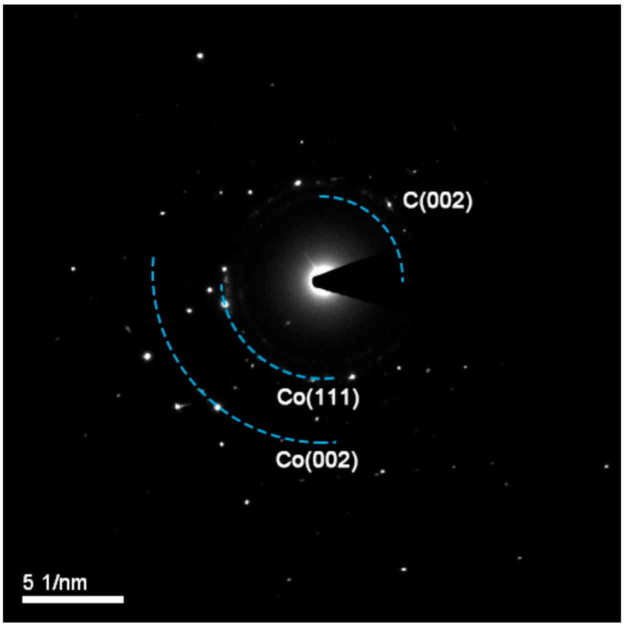

Figure S6. The SAED image of ZIF/CNF-700

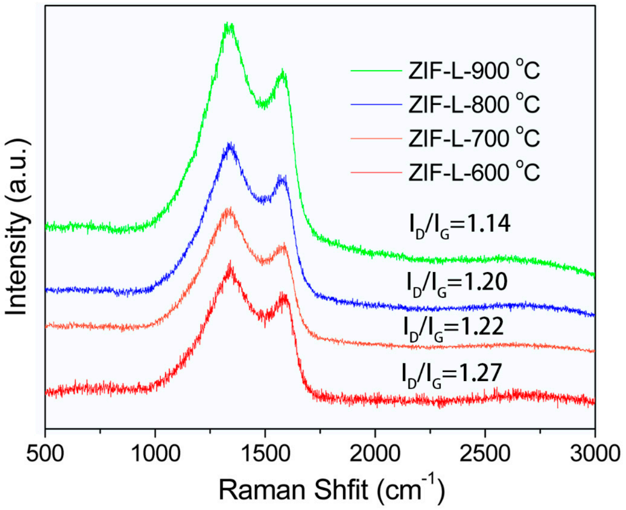

Figure S7. Raman spectra of ZIF-L/CNF with different annealing temperature.

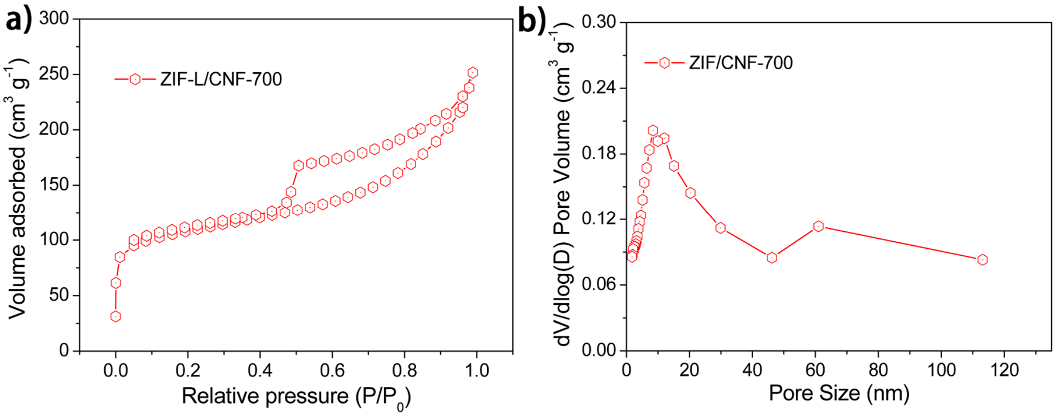

Figure S8. a) N_2 adsorption isotherms of ZIF/CNF-700 at 77 K; b) the corresponding pore size distribution.

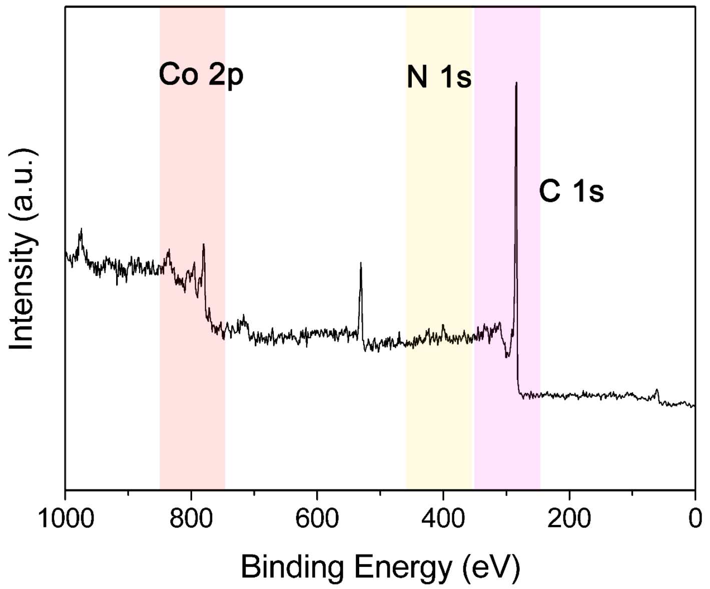

Figure S9. XPS survey spectrum of ZIF/CNF-700.

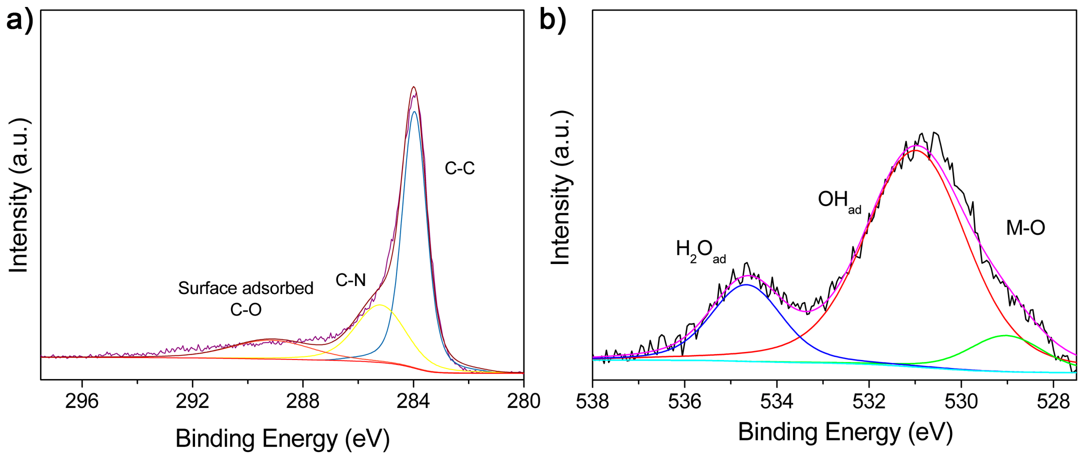

Figure S10. XPS a) C 1s and b) O 1s spectra of ZIF-L/CNF-700.

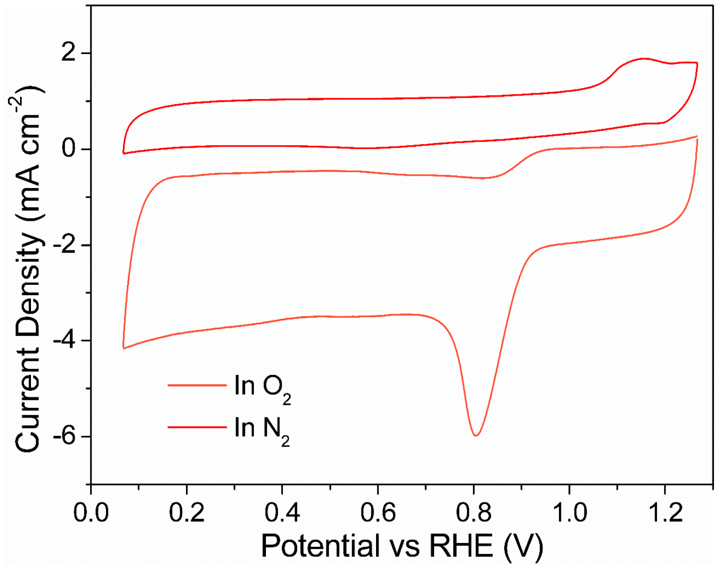

Figure S11. a) CVs obtained in O_2-saturated and N_2-saturated 0.1 m KOH.

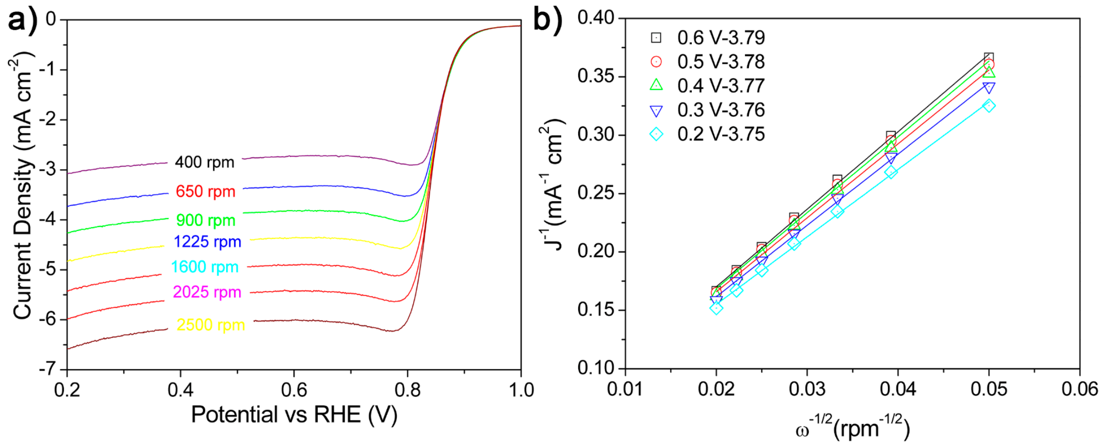

Figure S12. a) ORR polarization curves of ZIF-L/CNF-700 at different rotating speeds; b) the corresponding K-L plots of electron transfer numbers.

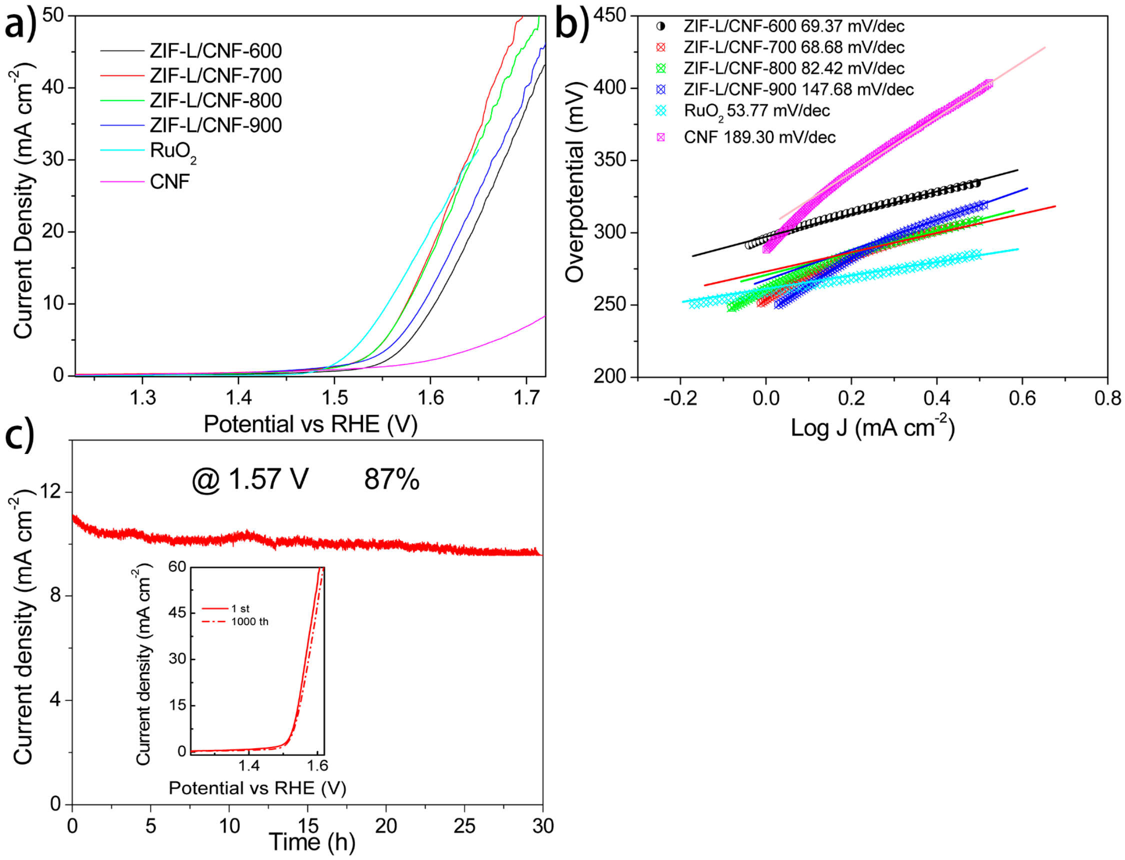

Figure S13. a) OER polarization curves of ZIF-L/CNF-700 and RuO_2 in 1.0 M KOH with a scan rate of 5 mV s^{-1}; b) The corresponding Tafel slopes; c) The OER polarization curves (inset) of ZIF-L/CNF-700 before and after the 10-h chronoamperometric i-t test.

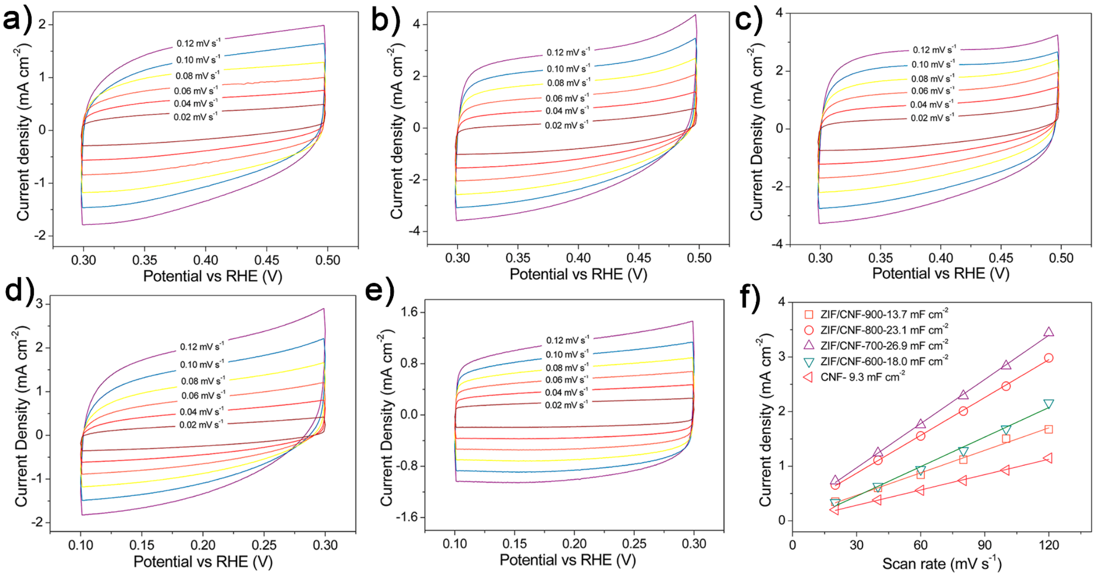

Figure S14. Cyclic voltammograms recorded at various scan rates in the non-Faradaic region in 1 M KOH for the ZIF-L/CNF-X catalysts. a) ZIF-L/CNF-900; b) ZIF-L/CNF-800; c) ZIF-L/CNF-700; d) ZIF-L/CNF-600 and e) CNF. f) The current density as a function of scan rate for all ZIF-L/CNF-X catalysts.

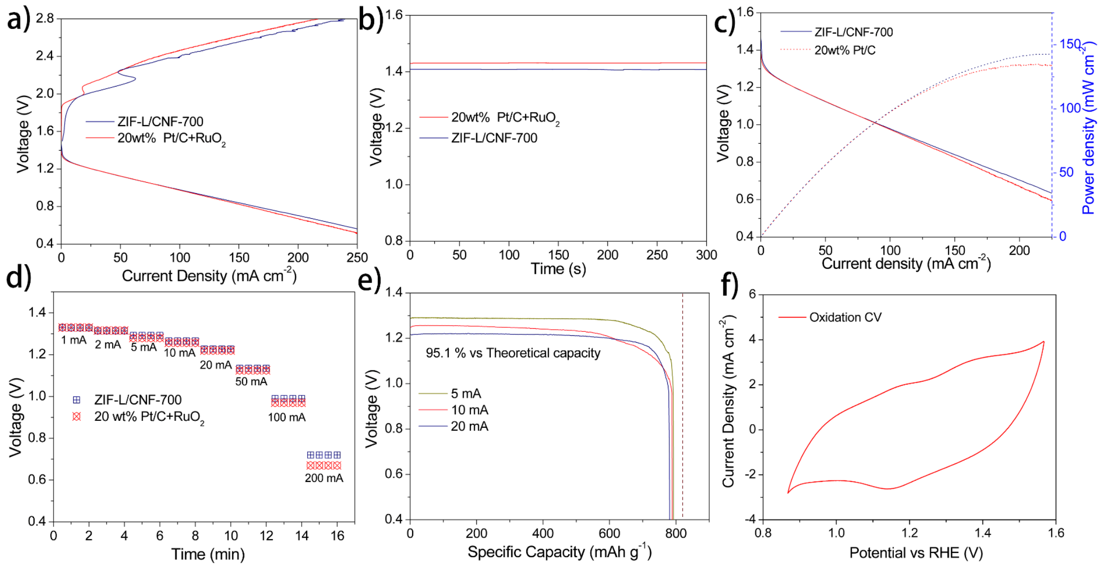

Figure S15. Performance of the aqueous ZABs using the bifunctional ZIF-L/CNF-700 as the air cathode catalyst. a) Polarization curves of charge and discharge for ZIF/CNF-700 and 20wt% Pt/C + RuO_2 benchmark; b) The ZAB open-circuit voltages; c) The corresponding power density plots; d) Battery voltage curves at different discharge rates; e) Battery capacities under various discharge rates for ZIF-L/CNF-700; f) The redox peak in the range of 0.85 V to 1.55 V, suggesting the catalyst itself can be oxidized and reduced.

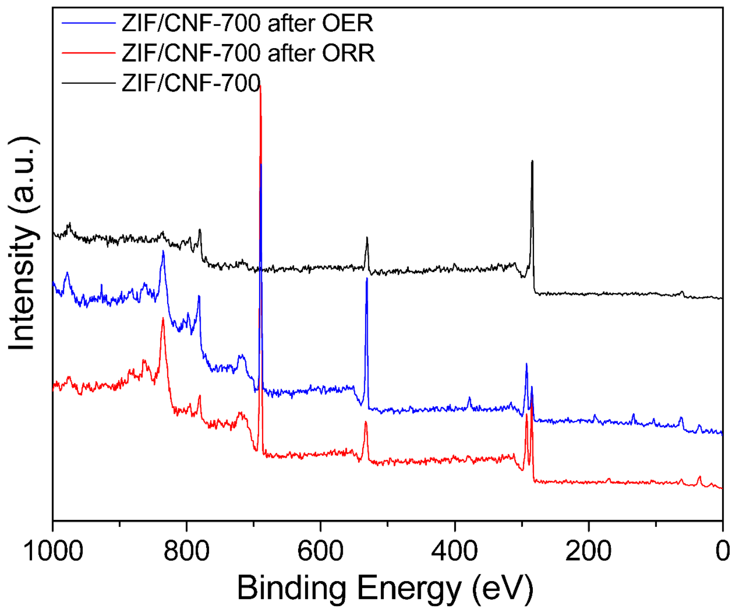

Figure S16. XPS survey spectrum of ZIF/CNF-700 before and after OER and ORR.

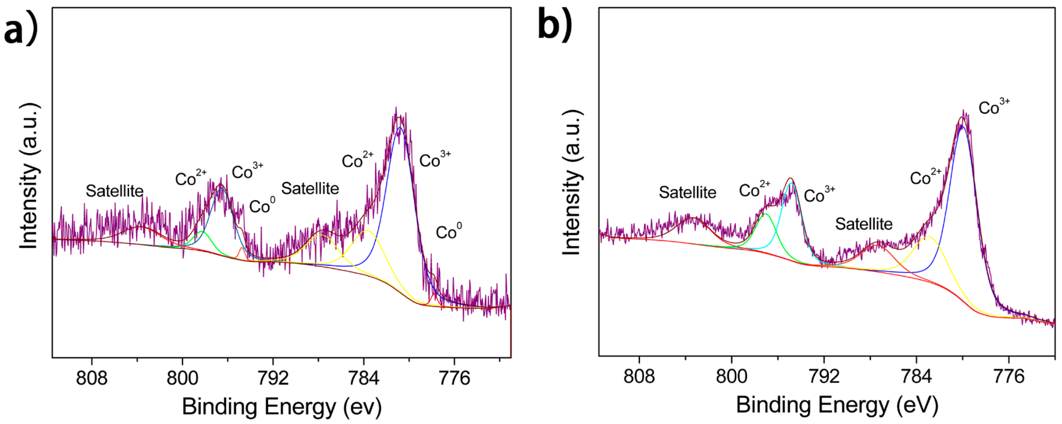

Figure S17. XPS Co 2p spectra of ZIF-L/CNF-700 a) after OER and b) after ORR.

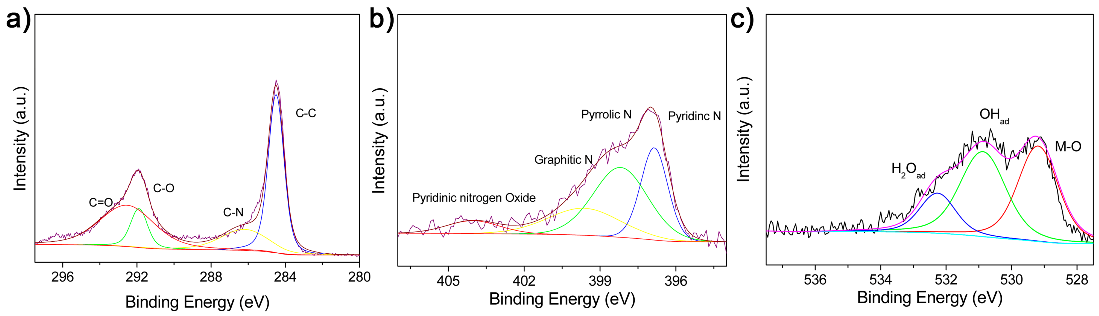

Figure S18. XPS a) C 1s b) N 1s and c) O 1s spectra of ZIF-L/CNF-700 after OER.

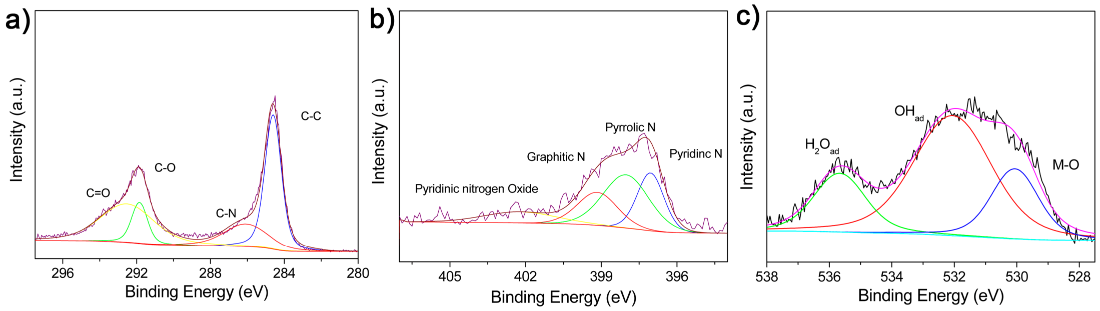

Figure S19. XPS a) C 1s, b) N 1s and c) O 1s spectra of ZIF-L/CNF-700 after ORR.
